# Do Superordinate Identification and Temporal/Social Comparisons Independently Predict Citizens’ System Trust? Evidence From a 40-Nation Survey

**DOI:** 10.3389/fpsyg.2021.745168

**Published:** 2021-11-05

**Authors:** Luca Caricati, Chuma Kevin Owuamalam, Chiara Bonetti

**Affiliations:** ^1^University of Parma, Department of Humanities, Social Sciences and Cultural Industries, Parma, Italy; ^2^Division of Organisational and Applied Psychology, University of Nottingham Malaysia, Selangor, Malaysia

**Keywords:** system justification, social identity, national identification, social comparison, temporal comparison

## Abstract

Do superordinate in-group bias as well as temporal and social comparisons offer standalone explanations for system justification? We addressed this question using the latest World Value Survey (7th Wave), combining the responses of 55,721 participants from 40 different nations. Results from a random slope multilevel model showed that superordinate (national) identification, temporal comparison (i.e., the outcomes of an individual relative to those of his/her parents at different time points), and social comparison (based on income levels) were *independent* and *positive* predictors of system justification. Specifically, system justification increased when national identification was high, when income increased (i.e., the socioeconomic comparison was positive), and when the outcomes of citizens improved relative to the outcomes of their parents at relevant time points (i.e., the temporal comparison was positive). Incidentally, we also observed an interaction between national identification and temporal comparison (but not with social comparison), indicating that positive temporal comparison seemed to have a reduced effect (but still significant) for highly identified citizens. These results are supportive of the social identity approach to system justification and suggest that support for societal systems is a positive function of people’s personal and group interests.

## Introduction

“*Beggars do not envy millionaires, just other beggars who are more successful*”

(Bertrand Russell)

Many people live within unequal social situations that they are often reluctant to challenge and are sometimes ardent supporters of these realities, even when it goes against some of their vested material or symbolic interests. Why is this so? According to the System Justification Theory (SJT; Jost and Banaji, 1994), this happens because people possess a specific “system justification motivation” to pursue the bigger picture (i.e., in believing that the system within which they operate is just and fair). This new motivation is assumed to sit beside the more traditional ego justification (i.e., the need to achieve a positive self-esteem) and group justification (i.e., the need to achieve a positive social identity) needs, and that it drives people to see societal arrangements, and its inequality, as the way that things should be (and, by so doing, ultimately legitimize the *status quo*). Importantly, system justification is assumed to satisfy existential, epistemic, and relational needs, permitting the reassuring belief that the world is a predictable, certain, and (relatively) safe place (Jost and Hunyady, 2005; Jost, 2019). Thus, according to SJT, the reason why people might be reluctant to challenge unequal social arrangements is that this would be extremely costly to the predictability and stability of realities to which they have become accustomed (Jost and Banaji, 1994; Jost and Hunyady, 2003).

Although most people possess this system justification motive (i.e., it is present for those who are advantaged and disadvantaged by the relevant systems), it is often easier for the advantaged to accommodate their ego-, group-, and system-based needs than for the disadvantaged because, for the former, these interests align. For the disadvantaged, however, meeting the demands of the system motive can generate some difficulty, often because supporting the relevant societal system tends to come at the expense of relegating their ego and/or group justification needs to the background (Jost and Banaji, 1994; Jost et al., 2004). It was for this reason that the classic SJT acknowledged (based on social identity principles) that the justification of societal systems should increase as social advantage also increases (Jost and Hunyady, 2005; Jost et al., 2012). Put simply, the advantaged ought to be more inclined to support a relevant societal system, given their privileged position within it, while the disadvantaged should (ordinarily) be reluctant to do so outside of the system justification motive. When this system motive is operational for the disadvantaged, SJT assumes that this will cause them to intensify their support for their social systems, especially if their ego and group justification needs are sufficiently subdued (Jost et al., 2003; Jost, 2017). But is this the only (or even the most plausible) explanation for system justification among the disadvantaged?

### An Alternative Explanation of System Justification

The idea that a specific motivation to justify the system is required to explain instances of system justification, beyond personal/group interest, has been challenged by Owuamalam et al. (2018, 2019a, 2019b) in their Social Identity Model of System Attitudes (SIMSA). SIMSA assumes that system justification, especially among the disadvantaged, can be explained by the traditional motives of personal/group interests, without recourse to an independent system motivation explanation. For example, SIMSA assumes that some instances of the puzzling justification of disadvantageous systems, sometimes seen among members of low-status groups, can result from them paying attention to their identity needs but at a more inclusive level of social categorization (e.g., their nation). So, for instance, African-Americans may justify disadvantageous realities in America, if their attention is strongly focused on the needs that are tied to their superordinate identity as Americans rather than the needs that are tied to their subgroup (African) identity. Therefore, African-Americans may justify disadvantageous systems (e.g., the American government) that regulate/oversee the institutional huddles confronting fellow group members (e.g., fatal law enforcement), if their attention is narrowly focused on their national (superordinate) identity as Americans. A similar process should also operate for high-status groups. In this sense, system justification is likely nothing more than a favorable evaluation of one’s superordinate in-group.

But, this superordinate in-group bias explanation is not the only one on offer under the social identity umbrella (i.e., social identity theory (SIT); Tajfel and Turner, 1979), especially given that SIMSA and its predecessor (SJT) do not currently say much about system justification of members of groups that are placed in an intermediate position (i.e., those who are disadvantaged but can nevertheless realize downward comparison) relative to those who are clearly advantaged or disadvantaged. This is the vacuum that the Triadic Social Stratification Theory (TSST, Caricati, 2018; Caricati and Owuamalam, 2020) fills, also drawing from SIT (Tajfel and Turner, 1979).

In particular, TSST offers one distinct reason why people might support their social systems, especially the puzzling instances of system justification among the disadvantaged. TSST, similar to its parent framework (SIT), assumes that people are motivated by a need for positive self-worth to improve their social position both personally (i.e., by upward individual mobility) and collectively (by upward social mobility) and that sometimes this goal can be reached by comparing the outcomes of an individual (or the outcomes of an individual’s social group) with those of others. Evidence shows that people are often motivated to enhance their own social position by upward *individual* mobility unless this goal is impeded in some way (Wright et al., 1990; Ellemers, 1993; Ellemers et al., 1993; Jackson et al., 1996). So, for example, an African-American may choose to compare him/herself with other in-group members (even their immediate family) who are not doing so well and may embrace America and consequently support its systems simply because it has afforded him/her the opportunity to rise above his/her parents or other members of their African-American community. Beyond the foregoing *social* comparisons, it is also possible for individuals to compare their outcomes against different time points in their life, so that a favorable comparison is achieved when individuals believe that they are doing better now than they did in the past (i.e., temporal comparison, Blanz et al., 1998).

TSST is, therefore, currently unique in its emphasis on the social comparison provision of SIT, arguing that so long as a given social or temporal stratification allows for intermediate positioning (whether it be within groups or between time points), that people may be motivated by the need for positive self-worth to support the *status quo* in which this was made possible. That is, they do so because (1) they are better off than others (individuals or groups) and (2) they are better off *now* than they were in the *past*.

Accordingly, it has been shown that people are more likely to justify their societal systems (Caricati and Sollami, 2018) and are even less likely to question these realities (Becker, 2012) when the *status quo* permits a downward comparison. Similarly, a comparison between actual and past conditions (Mummendey et al., 1996, 1999; Zagefka and Brown, 2005) has been suggested as an important factor when considering the extent to which people support their social systems. For example, people who successfully realize (or believe in) individual mobility may be more likely to support existing social arrangement and, consequently, also more likely to deny that prejudice or discrimination exists (Ng and Chiu, 2001; Derks et al., 2011, 2015; Owuamalam et al., 2017). Similarly, people who believe that their standard of life has improved with respect to the past may be motivated to justify the system that allowed for such improvement to materialize (Caricati and Owuamalam, 2020).

### Aims and Hypotheses

Although research has shown that social and temporal comparisons [as well as superordinate (national) identification] are able to bolster system support, none of these previous studies have examined, so far, these variables together, meaning that their unique contributions with regard to system justification have yet to be determined. This is important for two reasons at least. On the one hand, the national in-group provides the context to appraise the life outcomes of an individual relative to fellow nationals (including family members). From this perspective, it is possible to argue that a downward social comparison with other nationals could enhance the extent to which an individual observes (and appreciates) the benefits of greater (psychological) investments in a national in-group that made this favorable (self-worth-boosting) comparison possible. Therefore, in this situation, system support could be due to social comparison alone, or it could be due to its joint action with national identification. System support could also be due to “love of country” and not due to a favorable social/temporal comparison. In short, understanding the unique contribution of these explanations is difficult, if both are not simultaneously accommodated within the same model, to allow the partitioning of variances in system support that is due to each explanation.

At the same time, it is also possible that “love of country” (i.e., superordinate identification) could bolster entitlement feelings, and such feelings could poison the normally self-enhancing effect of downward temporal comparisons (e.g., when white working-class Americans compare their outcomes at present to what it was in the past, amidst the influx of competing migrant groups). From this perspective, then, it is possible that the ordinarily positive effect of favorable temporal comparison on system justification might cease (or be suppressed) when superordinate identification is taken into account. No other investigation has systematically unpacked these processes by considering them in tandem. Therefore, to be more certain that the social and temporal comparisons of TSST, as well as the superordinate in-group bias explanation of SIMSA are independent influencers of the system justification effect, one should demonstrate that they offer unique insights when considered together. In this investigation, therefore, we focused on three key self/group-interested predictors of system justification, namely, superordinate (national) identification (as per SIMSA), social comparison, with income as an indicator of relative social advantage, and temporal comparison (as per TSST).

First, we expected that system justification would be positively related to national identification over and beyond the alternative explanations (i.e., social/temporal comparisons) because, according to SIMSA, people would be more likely to support the national system to the extent that they identify with their nation (Hypothesis 1). Second, based on TSST, we expected that all otherwise being equal, the advantaged (as well as the intermediately positioned) would be more likely than their relatively more disadvantaged counterparts to justify the system (Hypothesis 2). This is because, in this situation, such individuals can obtain positive personal and group comparisons from a system that enabled their relatively advantaged social position. Finally, based on TSST, we considered the consequence of comparing own standard of living to those of one’s parents over time, which we used to proxy temporary comparison. It is important to note that this instance of intragroup comparison is consistent with evidence that people tend to prefer intragroup comparison over intergroup comparison and often engage in comparison with past outcomes as a means of dealing with social identity-based challenges (Major and Forcey, 1985; O’Brien and Major, 2009; Akfirat et al., 2016). Therefore, we expected that system justification would increase when people believe that they are better off at present than in the past, especially when this comparison is tied to those people that one ordinarily look up to (e.g., one’s parents, Hypothesis 3).

## Materials and Methods

### Sample

We used the 7th Wave of the World Value Survey (WVS) with 70,867 participants from 49 countries worldwide. We considered only participants with no missing values^1^ on the measures that were relevant to our analysis (refer to the explanation below), and this consideration reduced both the *N*-size (now down to 55,721 participants) and the number of nations (down to 40 countries: 47.7% men, mean age = 42.16, SD = 15.97, range = 16–103; Table 1).

**TABLE 1:** Sample demographic details and descriptive statistics for key and contextual variables.

		**System justification**		**Identification**		**Income**		**Comparison**			**Age**	**Education**	**GINI**	**GDP PPP ($)/1000**
					
	***N***	***M***	***SD***	***M***	***SD***	***M***	***SD***	**Same (%)**	**Better off (%)**	**Worse off (%)**				
Argentina	933	1.90	0.60	3.15	0.75	5.08	1.60	54.2	24.4	21.3	42.62	2.75	41.40	22.95
Australia	1,650	2.23	0.59	3.37	0.70	5.19	2.10	28.9	58.2	12.8	53.70	4.68	34.40	53.32
Bangladesh	1,182	2.87	0.65	3.29	0.74	5.63	2.08	5.0	86.4	8.6	36.62	1.86	32.40	4.95
Bolivia	1,970	1.80	0.59	3.37	0.83	5.03	2.02	51.7	39.6	8.7	37.86	3.41	42.20	9.09
Brazil	1,511	1.79	0.67	2.65	0.93	4.04	2.13	17.3	68.2	14.5	43.56	2.82	53.90	15.26
Myanmar	1,198	2.84	0.71	3.03	0.85	4.70	2.01	30.0	49.5	20.5	40.41	2.38	30.70	5.36
Chile	919	1.97	0.64	3.53	0.71	4.72	1.70	26.4	60.7	12.8	45.10	3.86	44.40	25.15
China	2,950	3.29	0.53	3.26	0.66	4.15	1.85	9.9	88.8	1.3	44.57	2.83	38.50	16.78
Colombia	1,498	1.79	0.60	3.16	0.82	4.43	2.53	51.2	37.2	11.6	38.87	3.12	50.40	15.64
Cyprus	823	2.15	0.71	3.41	0.80	5.20	1.71	21.1	58.9	19.9	45.10	4.28	31.40	41.25
Ecuador	1,138	1.97	0.67	3.17	0.72	4.74	2.20	66.1	24.2	9.8	39.43	3.20	45.40	11.85
Ethiopia	1,190	2.55	0.81	2.93	0.91	4.38	2.25	15.6	63.6	20.8	31.84	2.02	35.00	2.31
Germany	1,431	2.46	0.60	3.34	0.64	5.20	1.68	27.0	61.7	11.3	50.93	4.10	31.90	56.05
Greece	1,122	1.89	0.57	3.58	0.61	4.56	1.77	16.0	56.4	27.6	50.98	3.26	34.40	31.40
Guatemala	1,100	1.65	0.58	2.94	0.82	5.99	2.02	27.7	61.9	10.4	33.51	4.16	48.30	9.00
Indonesia	3,169	2.73	0.66	3.01	0.90	4.24	2.41	26.0	68.3	5.7	39.94	2.27	39.00	12.30
Iraq	1,156	1.77	0.75	3.58	0.86	4.46	1.83	41.3	21.5	37.3	36.59	2.84	29.50	11.33
Japan	1,044	2.47	0.58	3.28	0.66	4.27	2.72	31.5	48.6	19.9	56.42	4.43	32.90	43.24
Kazakhstan	1,058	2.81	0.71	3.26	0.75	5.53	1.68	29.3	56.0	14.7	41.83	4.82	27.50	27.44
South Korea	1,245	2.29	0.55	3.24	0.67	4.84	1.38	24.9	67.6	7.5	45.63	4.12	31.60	43.03
Kyrgyzstan	1,154	2.26	0.78	3.57	0.63	5.07	2.18	28.1	61.5	10.4	41.37	4.45	27.70	5.47
Lebanon	1,184	1.94	0.59	3.70	0.67	5.53	1.82	31.8	42.9	25.3	40.91	3.69	31.80	15.33
Malaysia	1,311	2.45	0.68	3.00	0.84	4.60	2.05	30.1	56.4	13.6	38.32	3.36	41.00	29.53
Mexico	1,699	1.65	0.68	3.42	0.82	4.22	2.38	27.7	57.7	14.6	43.19	3.04	45.40	20.41
Nicaragua	1,199	1.89	0.83	3.00	0.83	4.58	2.52	44.3	43.1	12.6	35.15	2.74	46.20	5.63
Pakistan	1,827	2.54	0.85	3.68	0.69	4.41	2.30	17.9	55.4	26.7	35.58	2.17	33.50	4.88
Peru	1,350	1.44	0.56	3.47	0.76	4.98	1.91	34.2	59.1	6.7	40.23	3.34	42.80	13.38
Philippines	1,198	2.91	0.63	3.30	0.65	4.40	2.08	49.8	41.6	8.6	43.71	2.34	44.40	9.28
Romania	1,047	1.82	0.67	3.34	0.72	5.41	1.95	26.1	58.5	15.4	48.01	3.24	36.00	32.30
Russia	1,608	2.34	0.76	3.08	0.83	4.79	1.93	34.6	46.4	19.0	45.73	4.85	37.50	29.18
Serbia	932	1.84	0.67	3.14	0.77	4.75	1.93	32.6	30.0	37.3	46.94	5.16	36.20	18.99
Vietnam	1,190	3.24	0.51	3.22	0.65	5.11	1.53	6.9	90.3	2.8	37.93	3.23	35.70	8.37
Zimbabwe	1,198	2.32	0.86	3.43	0.80	3.46	2.18	14.9	30.3	54.8	39.12	2.45	44.30	2.95
Tajikistan	1,177	3.18	0.66	3.54	0.73	5.63	1.59	17.9	67.4	14.7	41.21	4.26	34.00	3.52
Thailand	1,367	2.61	0.67	2.53	0.96	4.74	1.77	29.3	54.2	16.5	45.90	2.17	36.40	19.23
Tunisia	1,163	1.75	0.64	3.66	0.63	4.72	2.02	15.0	53.7	31.4	43.07	2.56	32.80	11.20
Turkey	2,260	2.73	0.68	3.23	0.76	5.34	1.72	27.7	45.8	26.5	38.83	2.35	41.90	27.88
Ukraine	1,130	1.81	0.69	3.19	0.73	4.46	1.92	25.7	52.4	21.9	47.90	4.93	26.10	13.34
Egypt	935	1.44	0.59	3.89	0.41	5.13	1.35	16.9	35.8	47.3	39.02	2.86	31.50	12.25
United States	2,505	2.11	0.54	2.97	0.83	5.04	1.88	32.1	46.7	21.1	43.62	4.89	41.40	65.28

### Outcome Variable

**System justification** has been operationalized in many ways by its principal proponent, such as out-group favoritism (Jost et al., 2004), general and economic system justification (Jost and Thompson, 2000; Kay and Jost, 2003), and trust/confidence in government (Jost et al., 2003) among others. In this study, we focused on the last operationalization (i.e., trust in government), which we assessed with four items asking participants to indicate the extent to which they were confident in the institutions of governance of their society, namely, parliament, government, political parties, and justice system/courts (1 = *a great deal*, 4 = *not at all*, reverse scored). We focused on trust in government (and its apparatuses) because it satisfies several auxiliary conditions that should enable the system motive to manifest. Because this system motive is theorized to be in conflict with the personal/group interests of people in disadvantaged groups (Jost et al., 2003, 2004), it should create an obstacle for our personal/group-interested predictions to operate. Specifically, systems of governance are institutions that objectively high-, intermediate-, and low-status people are often highly dependent on, also because these entities are stable and inescapable realities of citizens’ existence (Kay et al., 2009; Friesen et al., 2019; Jost, 2019). The inability to escape governments that regulate sub-systems that undermine people’s outcomes could cause a sense of personal control to decrease (Kay and Friesen, 2011; Laurin et al., 2013), and these situations should allow the system motive to take a prime position, while personal and group motives should be relegated to the rear position (based on SJT), meaning that it should be more difficult to find supportive evidence for the interest-based predictions derived from SIMSA and TSST, especially for low- and intermediate-status groups. A multilevel confirmatory factor analysis revealed that the four items on this scale had adequate reliability both within (*a* = 0.82) and between nations (*a* = 0.97). Items were then averaged so that higher scores indicate higher system justification (i.e., trust in governance).

### Predictors

**National identification** was measured with a single item asking participants “how close do you feel to your country?” (1 = *very close*, 4 = *not close at all*), which was reversed so that higher scores indicated high levels of national identification.

**A within-group temporal comparison** was measured with a single item asking participants “comparing your standard of living with your parents’ standard of living when they were about your age, would you say that you are better off, worse off, or about the same?” (1 = *better off*, 2 = *worse off*, and 3 = *about the same*).

**An income-based social comparison** was measured with a single item asking participants “On this card is an income scale on which 1 indicates the lowest income group and 10 the highest income group in your country. We would like to know in what group your household is. Please, specify the appropriate number, counting all wages, salaries, pensions, and other incomes that come in.”

### Control Variables

To control for the effect of other levels of social advantage and being, thus, able to better estimate the effect of variables of interest to our test, we added some level-1 and level-2 covariates. At level-1, we considered gender, age, and the higher level of education attained by participants (0 = Early childhood education, 8 = Doctoral or equivalent)^2^. At the country level, we considered the GINI index and GDP PPP (Gross Domestic Product based on Purchasing Power Parity) to account for the potential effect of objective wealth inequality in the nation (higher GINI indicates higher inequality) and national wealth level (both GDP and GINI were taken from the WVS database).

### Analysis

We performed a series of multilevel hierarchical models in which country was the nesting variable. Model 1 considered income (i.e., social comparison) and national identification as the predictors of system justification (i.e., trust in national governance). In Model 2, we added temporal comparison as a predictor. The income-based social comparison was coded using two dummy variables considering “the same” as a reference category. Model 3 added interactions between national identification and temporal and social comparisons to control for potential interactive effects. In all models, level-1 continuous variables were centered within nations while level-2 covariates were grand-mean centered. The slopes of all level-1 predictors were allowed to have random variation across nations, while covariates were treated as fixed effects. Analyses were performed with restricted maximum likelihood estimation using the lme4 package (Bates et al., 2015) in R (R Core Team, 2021).

## Results

### Preliminary Analysis

Table 2 reports both level-1 and level-2 zero-order correlations and descriptive statistics of measured constructs. At the individual level (refer to the correlation coefficients above the diagonal), the resulting associations between the principal variables were generally weak in magnitude but in the expected direction. Notably, system justification was positively correlated with (1) positive temporal comparison, (2) national identification, and (3) income. With respect to the control variables: women, older and less educated people appeared to be more likely to justify the system.

**TABLE 2:** Zero-order correlations and descriptive statistics for variables at level-1 (upper triangle) and level-2 (lower triangle).

	*M (National level)*	*SD*	1	2	3	4	5	6	7
1. System justification	2.24	0.50	–	0.16**	0.05**	0.01*	0.01*	0.01*	−0.10*
2. Temporal comparison^	1.35	0.25	0.48**	–	0.03**	0.14**	0.00	0.01	0.04**
3. National identification	3.27	0.28	–0.19	–0.24	–	0.04**	−0.03**	0.08**	0.02**
4. Income	4.82	0.51	0.01	0.22	0.15	–	−0.03**	−0.12**	0.26**
5. Sex (0 = Male)	0.52	0.04	0.10	0.32*	–0.14	0.06	–	−0.02**	−0.04**
6. Age	42.28	5.31	–0.02	0.04	0.06	–0.07	0.48**	–	−0.13**
7. Education	3.38	0.95	–0.17	0.01	0.07	0.33*	0.39**	0.50**	–
8. GINI	37.55	6.70	–0.25	0.00	−0.40**	–0.27	–0.21	–0.25	–0.26
9. GPD PPP ($)/1000	20.15	15.64	–0.06	0.06	–0.07	0.18	0.13	0.68**	0.54**
*M (Individual level)*			2.29	1.38	3.25	4.78	0.52	42.16	3.32
x*SD*			0.83	0.76	0.81	2.07	0.50	15.97	2.01

*p < 0.01; **p < 0.001; ^ 0 = worse off, 1 = the same, 2 = better off. *N* for upper diagonal = 55,721; *N* for lower triangle = 40.

### Hierarchical-Level Modeling

Supporting the use of multilevel modeling, a null model in which only the intercept varied randomly across nations revealed that 36.4% of the variance in system justification was due to the different nations represented in the survey [Intraclass correlation (ICC) = 0.364; χ^2^(1) = 25638.00, *p* < 0.001].

Table 3 depicts results from the estimated models. First, models indicated that people, on average, did not express much trust in the system of governance of their nation (i.e., their level of system justification was low). More importantly, as indicated, both income and national identification were significantly and positively associated with system justification in all models. Model 2 indicated, as expected, a main effect of temporal comparison *F*(2, 37) = 21.22, *p* < 0.001 so that those who believed that their situation has worsened now than what it was in the past justified the system significantly less (*M* = 2.13, SE = 0.072) than people who believed that their social condition was the same (at least relative to their parents, *M* = 2.22, SE = 0.075). Moreover, those who believed that their situation has somewhat stagnated (i.e., “the same” group) were also less likely to justify the system compared with those who reported being better off now than in the past (*M* = 2.26, SE = 0.079).

**TABLE 3:** Fixed effects of model estimations.

	Null model	Model 1	Model 2	Model 3
	
	*b* (SE)	*b* (SE)	*b* (SE)	*b* (SE)
Income [cwc]		0.017 (0.005)**	0.013 (0.005)**	0.013 (0.005)**
Temporal comparison: The same vs. worse off (D1)			−0.089 (0.015)***	−0.090 (0.015)***
Temporal comparison: The same vs. better off (D2)			0.041 (0.015)**	0.041 (0.015)**
National identification [cwc]		0.091 (0.012)***	0.088 (0.011)***	0.100 (0.013)***
Identification × Income				0.002 (0.002)
Temporal comparison (D1) × Identification				−0.031 (0.011)**
Temporal comparison (D2) × Identification				−0.011 (0.009)
Sex [0 = Male]		0.017 (0.006)**	0.017 (0.006)**	0.017 (0.006)**
Age [cwc]		0.000 (0.000)	0.000 (0.000)	0.000 (0.000)
Education level [cwc]		−0.022 (0.002)***	−0.021 (0.002)***	−0.021 (0.002)***
GINI [gmc]		−0.022 (0.011)^	−0.019 (0.010)^†^	−0.018 (0.010)^†^
GDP PPP ($)/10000 [gmc]		−0.026 (0.048)	−0.032 (0.045)	−0.032 (0.045)
Intercept	2.24 (0.079)	2.21 (0.078)	2.21 (0.075)	2.21 (0.075)
N	55,721	55,721	55,721	55,721
ICC	0.36	0.37	0.36	0.37
AIC	111786.40	110282.80	109885.80	109908.30
BIC	111813.20	110416.70	110118.00	110167.30

cwc, centered within clusters; gmc, grand-mean centered. ^†^*p* < 0.09, ^ *p* = 0.056, **p < 0.01, ***p < 0.001. Model 1: predictors were income and national identification; Model 2: temporal comparison (dummy coded, D1 and D2) was added as a predictor; Model 3: interactions between national identification and social and temporal comparison were added.

Considering covariates, results indicated that women justified their national systems of governance more than men did. Interestingly, more educated people were *less* (not more) likely to justify their societal systems of governance. GDP (i.e., the objective index of societal wealth) and GINI (i.e., the objective index of societal-level inequality) appeared to have no significant main effects on system justification in the current data.

On an exploratory basis, we considered, in Models 3, the interactions between national identification and temporal and social comparisons, given our *a priori* speculation that national identification could actually fade (or suppress) the effects of social/temporal comparisons on system justification. Results revealed that national identification × income interaction was not significant (*b* = 0.002, SE = 0.002, *p* = 0.235), suggesting that social comparison is by-and-large a unique explanation for system justification that may not necessarily be contingent on superordinate identification (at least in this case). However, national identification interacted with temporal comparison to predict system justification, *F*(2, 43,0341) = 3.867. *p* = 0.021. When we decomposed this interaction by examining the association between temporal comparisons and system justification when superordinate (national) identification was high (*M* + 1SD) vs. low (*M*-1SD), we found, consistent with our speculation, that positive temporal comparisons seem to work best, in terms of its boosting effect on system justification, when national identification was low (Δ*b*_*same*–worse_ = 0.07, SE = 0.02, *p* = 0.0003; Δ*b*_*better*–same_ = 0.05, SE = 0.02, *p* = 0.006). Meanwhile, when superordinate (national) identification was high, positive temporal comparisons significantly predicted an increase in system justification also, but only in relation to the same vs. worse-off contrast (Δ*b*_*same–worse*_ = 0.11, SE = 0.02, *p* < 0.0001) and the better-off vs. worse-off contrast (Δ*b*_*better–worse*_ = 0.15, SE = 0.02, *p* < 0.0001), but not in relation to the better-off vs. same contrast (Δ*b*_*same–better*_ = −0.03, SE = 0.02, *p* = 0.120).

## Discussion

Do superordinate identification and social/temporal comparisons independently predict trust in systems of governance (i.e., system justification)? To answer this question, we simultaneously tested for unique contributions of distinct explanations from the social identity tradition (i.e., SIMSA and TSST). We wanted to observe whether the empirical evidence supports their theorized independence. Results were supportive of their theorized uniqueness: That is, as independent insights into the system justification phenomenon. Specifically, and as expected, national identification (as per SIMSA), temporal comparison, and income-based social comparison (as per TSST) independently predicted system justification along theorized lines, and accounting for either of these explanations did not obscure the visibility of the other accounts. In fact, national identification was positively associated with system justification despite accounting for social/temporal comparisons and vice versa.

In particular, the finding that favorable social comparisons boosted system justification conceptually replicates the studies by Caricati and Lorenzi-Cioldi (2012) and Caricati (2017) and indicates that people who benefit from the *status quo* are also more inclined to believe in (and support) that system. According to TSST, this might be because the possibility to find positive downward comparison becomes enhanced as income rises: That is, as the income of people increases, more opportunities for downward comparison becomes apparent, and therefore, the greater the potential for them to enhance their self-worth by looking at others who have not made it as far as they did.

Nonetheless, we acknowledged that the sizes of the effects that were detected in the current analyses were quite “tiny” (Cohen, 1988). This leaves open the possibility that system justification could also be affected by other variables beyond the ones that we set out to test. For example, results indicated that “country” explained a significant portion of the variance in system trust and this potentially suggests that the contextual conditions of national functioning, as well as culture-related factors, might jointly impact the level of trust of people in their national governments. In this study, we considered only two national factors that were relevant for the intent of the research [i.e., GINI index and gross domestic product based on purchasing power parity (GDP PPP)]. However, it is important to emphasize that we were limited in our use of the current secondary data to obtain appropriate measures of variables relevant to other SIMSA explanations (e.g., hope for future improvement, Bonetti et al., 2021; Owuamalam et al., 2021; and social reality caveats, Owuamalam et al., 2019a) and, for TSST, to directly test the applicability of the fear-of-falling assumption underlying system justification among intermediately positioned groups (Caricati and Owuamalam, 2020; Caricati et al., 2020). Future studies could address these shortcomings using primary data. Such future research could also incorporate other key assumptions underlying both SIMSA and TSST, concerning the manner in which the stability and legitimacy of social stratification could impact system-justifying attitudes (e.g., trust in government) of low- and intermediate-status groups.

Beyond the foregoing limitations, our results suggest, for the most part, that system justification can result from rational choices that people are making to support a system: (1) to which they feel connected and (2) which provides the opportunity to enhance self-worth by positive social and temporal comparisons. These results are important because they cast some doubt over the claim (elsewhere in the literature on SJT) that the system-justifying attitudes of the disadvantaged (including intermediately positioned ones) are irrational (Jost, 2019). It is to be recalled that the bifocal lens of SJT only recognizes the advantaged vs. disadvantaged and, from the standpoint that the system-justifying attitudes of disadvantaged are irrational (Jost, 2019), it would be tempting to conclude the system support of those disadvantaged people who are intermediately positioned in the income distribution, also does not make sense. But, in this study, we have shown that it does make sense because, similar to their wealthier counterparts, the middle (income) class people are uniquely positioned to experience not only the “lows” of the *status quo* (e.g., when the focus of comparison is upward) but also its “highs” (e.g., when the focus of comparison is downward). Therefore, disadvantaged people who are intermediately positioned in the status hierarchy (e.g., the middle class) are the ones, by virtue of their unique position, better able to notice that upward mobility is possible and, consequently, also the ones more likely to have a *realistic* hope that things will get even better in the future, and this can cause support for systems that permit this optimism to thrive (Owuamalam et al., 2021). In short, disadvantaged people in the middle of the income ladder can (and do) support the systems of governance of their nations. Such an orientation may not necessarily be because they are driven by an irrational system motive, but because there is ample opportunity to favorably compare their outcomes with others who are lower than they are in the income distribution (Caricati and Owuamalam, 2020), in manners that provide a realistic hope that future improvements to their outcomes in the existing system are also possible (Owuamalam et al., 2021). Worthy of note is the incidental temporal (but not social) comparisons by national identification interaction effect on system justification. Specifically, we found that positive temporal comparisons were best at boosting system justification for those who are weakly identified with their nation (i.e., system justification increases from negative temporal comparison to positive temporal comparison). Interestingly, however, and for those strongly identified with their nation, favorable temporal comparisons only boosted support for societal systems when the frame of reference concerned a point in time people felt that they were worse off than their parents (e.g., those who experienced improvement or stagnation of their social condition justified the system to the same extent). Thus, although a strong superordinate identification could soften the boosting effect of positive temporal comparisons on system justification as we had speculated, this trend seems to be specific to those instances where temporal contrasts were unlikely to have had a measurable boost in people’s self-worth (i.e., a comparison between better off vs. same is unlikely to matter much to self-worth [because the outcome deficit is narrower] compared to when the frame of reference is being worse off). However, we acknowledged the exploratory nature of the current findings, and future experimental studies could build on this initial correlational evidence to confirm whether elevated self-esteem/worth is, in fact, the mechanism that drives the boosting effect of positive temporal comparisons on system justification among people whose support for the *status quo* already benefits from a strong investment in their superordinate in-group.

### Limitation

This research, as any other correlational research, does not allow causal inference, and thus, some caution is needed in this respect. For example, it is possible that people who strongly trust their national institutions may identify strongly with their national in-group, rather than the opposite. However, when we examined this possibility, we found that a model in which national identification was the dependent variable and system justification (i.e., trust in governance) was the predictor, also produced a positive relationship, *b* = 0.118, SE = 0.02, *p* < 0.001, although the fit of this latter model was reliably poorer than the preferred reverse (i.e., Model 2), based on poorer fit indices (difference between Model 2 and alternative model: ΔAIC = −16948.90, ΔBIC = −16948.90, ΔICC = 0.24, Table 3). Note that even though this reverse causation is plausible, it would imply that a credible system provides individuals with a reason to identify with their nation, and this outcome will be more consistent with the rationality implied in the social identity perspective than with the irrationality implied in competing frameworks that assume system justification has less (if at all anything) to do with social identity needs.

## Conclusion

The fact that the disadvantaged people more or less tolerate societal systems that anchor the inequality that adversely affects them could be puzzling, especially in places where the assumption that people can change realities that do not work for them is strong (e.g., Western democracies). However, this puzzle begins to wane when consideration is given to the following:

a) The extent to which disadvantaged people take pride in, or identify with a superordinate (national) in-group that provides another source for positive social esteem;
b) The favorable social comparisons that could allow people to boost their sense of self-worth, especially those disadvantaged people who are intermediately positioned within the status hierarchy;
c) The favorable temporal comparison that allows the disadvantaged to feel worthy, even if their outcomes may not be as promising as those of individuals who come from more affluent backgrounds.

In the foregoing situations, our data shows that intermediately positioned people (either socially or temporally favored with regard to the outcome of the comparison, or simply by embracing their superordinate in-group) can trust and support existing societal arrangements because these realities meet their social identity needs. In short, we demonstrated that the superordinate in-group bias account as well as social and temporal comparisons offer unique insights into the system-justifying attitudes of the disadvantaged (especially those who are intermediately positioned).

## Data Availability Statement

Publicly available datasets were analyzed in this study. This data can be found here: https://www.worldvaluessurvey.org/WVSDocumentationWV7.jsp.

## Ethics Statement

Ethical review and approval was not required for the study on human participants in accordance with the local legislation and institutional requirements. Written informed consent for participation was not required for this study in accordance with the national legislation and the institutional requirements.

## Author Contributions

LC and CO contributed equally in drafting and revising the manuscript, data analysis, and data interpretation. CB involved in drafting and revising the manuscript. All authors contributed to the article and approved the submitted version.

## Conflict of Interest

The authors declare that the research was conducted in the absence of any commercial or financial relationships that could be construed as a potential conflict of interest.

## Publisher’s Note

All claims expressed in this article are solely those of the authors and do not necessarily represent those of their affiliated organizations, or those of the publisher, the editors and the reviewers. Any product that may be evaluated in this article, or claim that may be made by its manufacturer, is not guaranteed or endorsed by the publisher.

## Footnotes

1 On the whole, there were 21.3% of missing values of which 4.6% were on the dependent variable.

2 We planned to also include left-right political orientation (1 = left, 10 = right). However, this measure was not administered in nine countries and was missing for many participants, so that its inclusion would have decreased sample size to 36,918, representing an attrition rate of more than 30%. Given that political orientation was not a principal variable in this study and that its inclusion did not change results substantially, we decided not to include political orientation in our main analysis. For the sake of transparency, we also reported the results of the models with political orientation as covariate in Supplementary Tables 1, 2.
