# Supplementary material for: Do Superordinate Identification and Temporal/Social Comparisons Independently Predict Citizens’ System Trust? Evidence From a 40-Nation Survey
Source: Front Psychol. 2021 Nov 5;12:745168. doi: 10.3389/fpsyg.2021.745168 (PMC8603914; doi:10.3389/fpsyg.2021.745168)
Supplement: Supplementary file 1 [file Data_Sheet_1.PDF]

Supplementary material

Table S1. *Zero Order Correlations and Descriptive Statistics for Left-right political orientation.*

	Left-right orientation (Individual level, n = 36,918)	Left-right orientation (National level, n = 31)
1. System Justification	.15**	.59**
2. Temporal Comparison^	.04**	.31
3. National identification	.09**	.02
4. Income	.05**	.18
5. Sex (0 = Male)	-.01*	-.13
6. Age	.01*	-.44**
7. Education	-.10**	-.44**
8. Gini		-.06
9. GPD PPP ($)/1000		-.55**
M	5.83	5.81
SD	2.51	0.62

Note. * *p* < .01; ** *p* < .001. ^ 0 = worse off, 1 = the same, 2 = better-off.

Table S2. *Fixed effects of models with political orientation as covariate*

	Model 1	Model 2	Model 3
	b (*se*)	*b* (*se*)	*b* (*se*)
Income [cwc]	0.017 (0.006)**	0.014 (0.006)*	0.014 (0.006)*
Temporal Comparison			
The same vs. worse off (D1)		-0.090 (0.016)***	-0.092 (0.016)***
The same vs. better off (D2)		0.039 (0.017)*	0.039 (0.017)*
National identification [cwc]	0.077 (0.013)***	0.075 (0.013)***	0.086 (0.014)***
Identification x Income			0.001 (0.002)
Temporal comparison (D1) x Identification			-0.033 (0.013)*
Temporal comparison (D2) x Identification			-0.010 (0.010)
Sex [0 = Male]	0.020 (0.007)**	0.020 (0.007)**	0.020 (0.007)**
Age [cwc]	0.000 (0.000)	0.000 (0.000)	0.000 (0.000)
Education level [cwc]	-0.020 (0.002)***	-0.020 (0.002)***	-0.020 (0.002)***
Left-right political orientation [cwc]	0.017 (0.001)***	0.017 (0.001)***	0.017 (0.001)***
Gini [gmc]	-0.029 (0.012)*	-0.021 (0.010)†	-0.021 (0.007) †
GDP PPP ($)/10000 [gmc]	-0.027 (0.046)	-0.024 (0.040)	-0.025 (0.040)
Intercept	2.21 (0.077)	2.20 (0.075)	2.20 (0.075)
N	36,918	36,918	36,918
Countries	31	31	31
ICC	.31	.30	.31
AIC	72049.72	71753.89	71777.77
BIC	72185.98	71983.84	72033.27

cwc = centered within clusters; gmc = grand mean centered

† $p < .06$, * $p < .05$, ** $p < .01$, *** $p < .001$.

Model 1: predictors were income and national identification; Model 2: temporal comparison (dummy coded, D1 and D2) was added as predictor; Model 3: interactions between national identification and social and temporal comparison were added.
